# The relationship between the BRAF p.V600E mutation and a family history of CRC in the early-onset CRC cases from the Australasian Colon Cancer Family Study

**DOI:** 10.1186/1897-4287-10-S2-A23

**Published:** 2012-04-12

**Authors:** DD Buchanan, AK Win, R Walters, MD Walsh, M Clendenning, B Nagler, E Pavluk, SA Pearson, C Rosty, J Maskiell, JL Hopper, MA Jenkins, JP Young

**Affiliations:** 1 Familial Cancer Laboratory, Queensland Institute of Medical Research, Bancroft Centre, Herston QLD 4006, Australia; 2 Centre for MEGA Epidemiology, School of Population Health, University of Melbourne, Carlton VIC 3053, Australia; 3 Molecular and Cellular Pathology, University of Queensland, Herston, QLD 4006, Australia

## Background

The *BRAF* p.V600E somatic mutation is present in approximately 10-20% of unselected colorectal cancer (CRC) and 30% -75% of CRCs demonstrating high levels of microsatellite instability (MSI-H). Currently, testing for the *BRAF* p.V600E mutation is undertaken to exclude Lynch syndrome in MSI-H CRCs that demonstrate loss of the MLH1 and PMS2 proteins by IHC. However, recent evidence suggests that the *BRAF* p.V600E mutation is associated with a familial predisposition to serrated neoplasia, an increased risk of CRC and possibly extra-colonic cancers in relatives. The aim of this study was to determine 1) if early-onset CRC with the *BRAF* p.V600E mutation is associated with a family history of CRC, and 2) if pathological features of *BRAF* positive CRCs associate with CRC development in relatives.

## Methods

Population-based recruitment of probands into the Australasian Colon Cancer Family Study between 1997 and 2006 was undertaken for newly diagnosed CRC irrespective of any family history of cancer but limited to a first primary adenocarcinoma of the colon or rectum between the ages of 18-60yrs. Patients with Lynch syndrome and MutYH associated polyposis were excluded from the analysis. The *BRAF* p.V600E mutation was determined using a previously described allele-specific PCR assay on DNA from formalin-fixed paraffin embedded colorectal cancer tissue.

## Results

The average age of onset for the 709 probands (49.5% female) was 46.3yrs ± 7.9yrs (SD) with a range of 18yrs to 60yrs. A history of CRC in at least one first degree relative (FDR) or second degree relative (SDR) was reported in 39.5% (280/709) of the probands. The *BRAF* p.V600E mutation was present in 54/709 (7.6%) CRCs. Overall, probands with a *BRAF* p.V600E positive CRC were less likely to have a FDR with CRC than probands with a *BRAF* wildtype CRC (OR=0.42, 95%CI=0.16-1.09; P=0.07) regardless of MSI status. Among the probands with a *BRAF* p.V600E mutated CRC, the mean age at diagnosis was significantly older for probands with a CRC-affected FDR or SDR (50.4 years, 95%CI=47.1–53.7) when compared to probands without a family history (44.0 years, 95%CI=40.7–42.3; *P* = 0.02). The odds (risk) of having a family history of CRC significantly increased 11% per year of age (OR 1.11; 95% CI 1.01 – 1.21; P = 0.02) in probands with a *BRAF* p.V600E mutation positive CRC. When considering only *BRAF* p.V600E positive CRC, a mucinous histology was increased 4.4 fold in probands with a FDR or SDR with CRC (n=17) when compared to probands without a family history of CRC (n=37), although this was not significantly different (OR=4.40; 95%CI = 0.59 – 32.62; P=0.15).

## Conclusions

In this study of early-onset CRC, we describe evidence for a relationship between the *BRAF* p.V600E mutation and a family history of CRC that is related to an increasing age of CRC onset. These findings, in conjunction with observations suggesting certain pathological features are associated with a family history of CRC in *BRAF* p.V600E mutated CRC warrant further investigation.

